# Aroma-Driven Differentiation of Wuyi Shuixian Tea Grades: The Pivotal Role of Linalool Revealed by OAV and Multivariate Analysis

**DOI:** 10.3390/foods14132169

**Published:** 2025-06-21

**Authors:** Mengzhen Zhang, Ying Zhang, Yeyun Lin, Yuhua Wang, Jishuang Zou, Miaoen Qiu, Qingxu Zhang, Jianghua Ye, Xiaoli Jia, Haibin He, Haibin Wang, Qi Zhang

**Affiliations:** 1 College of Resources and Environment, Fujian Agriculture and Forestry University, Fuzhou 350002, China; zhangmengzhen1005@163.com; 2 College of Tea and Food Science, Wuyi University, Wuyishan 354300, China; 13559195845@163.com (Y.L.); a13883402655@163.com (J.Z.); jhye1998@126.com (J.Y.); jiaxl2010@126.com (X.J.); 3 College of Horticulture, Qingdao Agricultural University, Qingdao 266109, China; yingzhang@stu.qau.edu.cn; 4 College of JunCao Science and Ecology, Fujian Agriculture and Forestry University, Fuzhou 350002, China; 1220525008@fafu.edu.cn (Y.W.); miaoenqiu06@outlook.com (M.Q.); 2210525002@fafu.edu.cn (Q.Z.); 5 School of Marine Biology, Xiamen Ocean Vocational College, Xiamen 361100, China; alexhhb@163.com; 6 College of Life Sciences, Longyan University, Longyan 364012, China

**Keywords:** Wuyi Shuixian tea, odor activity value (OAV), flavor-active compounds, aroma profiling, quality grading

## Abstract

Wuyi Shuixian tea, a premium oolong tea known for its complex floral-fruity aroma, exhibits significant quality variations across different grades. This study systematically analyzed the aroma characteristics and key fragrant compounds of four grades (Grand Prize SA, First Prize SB, Outstanding Award SC, and Non-award SD) using headspace solid-phase microextraction-gas chromatography-mass spectrometry (HS-SPME-GC-MS), odor activity value (OAV) analysis, and multivariate statistical methods. A total of 159 volatile compounds were identified, with similar compound categories but distinct concentration gradients between grades. OAV-splitting analysis (based on OAV ≥ 1 as the threshold for aroma activity) identified β-ionone (fruity), octanal (fatty), and linalool (floral) as core aroma-active contributors, as their OAV values significantly exceeded 10 in awarded grades (SA, SB, SC), indicating dominant roles in sensory perception. Notably, linalool, a floral marker, showed a concentration gradient (SA > SB > SC) and was absent in SD, serving as a critical determinant of grade differentiation. Orthogonal partial least squares-discriminant analysis (OPLS-DA) further distinguished awarded grades (SA, SB, SC) by balanced fruity, floral, and woody notes, while SD lacked floral traits and exhibited burnt aromas. This classification was supported by hierarchical clustering analysis (HCA) of volatile profiles and principal component analysis (PCA). Electronic nose data validated these findings, showing strong correlations between sensor responses (W5S/W2W) and key compounds like hexanal and β-ionone. This study elucidates the molecular basis of aroma-driven quality grading in Wuyi Shuixian tea, providing a scientific framework for optimizing processing techniques and enhancing quality evaluation standards. The integration of chemical profiling with sensory attributes advances precision in tea industry practices, bridging traditional grading with objective analytical metrics.

## 1. Introduction

Oolong tea, distinguished by its semi-fermented processing and intricate floral-fruity aroma profile, occupies a prominent position in the global tea markets. Within this category, Wuyi Rock Tea (WRT) has earned international acclaim for its unique Yan Yun (“rock rhyme”)—a sensory hallmark originating from biogeochemical interactions within the mineral-rich terroir of the Wuyi Mountains. This characteristic is particularly pronounced in the Shuixian cultivar (*Camellia sinensis* cv. Shuixian), a nationally protected tea germplasm ranking second in cultivation scale among Wuyi tea varieties, surpassed only by Rougui [1,2,3]. The sensory superiority of WRT stems from its volatile organic compounds (VOCs), which, despite being only 0.01% dry mass, orchestrate consumer preference through threshold-dependent odor perception and synergistic interactions [4,5,6]. To date, more than 700 aroma compounds have been identified in tea, with oolong tea containing more than 300 distinct volatiles [7,8,9]. Of the many aroma components, only a few dozen play a leading role in the flavor of tea. For example, volatile monoterpenes and sesquiterpenes, such as geraniol, linalool, and nerolidol, mostly have flowers and fruits [10], and their content and composition play a key role in the formation of oolong tea aroma quality [11].

Tea aroma components are volatile, and the extraction process is susceptible to external conditions, so an effective aroma determination method is very important. Recent advancements in analytical techniques, particularly headspace solid-phase microextraction coupled with gas chromatography-mass spectrometry (HS-SPME-GC-MS), have revolutionized the quantification and identification of trace aroma compounds. This method enables precise detection of thermally labile volatiles while minimizing matrix interference, making it indispensable for tea aroma profiling [12,13,14]. Furthermore, the odor activity value (OAV), defined as the ratio of compound concentration to its odor threshold, has emerged as a robust metric for evaluating aroma contributions. Compounds with OAV > 1 are considered key drivers of sensory perception, providing a scientific basis for quality grading [15,16,17].

Despite progress, critical gaps persist in understanding how VOC profiles correlate with Wuyi Shuixian tea grades. Existing studies have focused primarily on general aroma characterization, ignoring systematic comparisons across quality tiers or mechanistic interpretations of grade-specific aroma markers [2,18,19]. Moreover, traditional evaluation methods heavily rely on subjective sensory panels, lacking objective chemical indicators for quality assurance. To address these limitations, this study integrates HS-SPME-GC-MS with multivariate statistical analysis (PCA, OPLS-DA) and the OAV-splitting method to: (1) Decipher VOC profiles across four Shuixian grades (SA, SB, SC, SD); (2) Identify grade-discriminatory aroma compounds; and (3) Establish quantitative relationships between odor characteristics and quality tiers. By bridging sensory evaluation with molecular insights, this work aims to advance precision in tea quality control and processing optimization.

## 2. Materials and Methods

### 2.1. Experimental Protocol

The experimental protocol was based on the comparison between the aromatic components of four different grades of Wuyi Shuixian tea provided by the Tea Bureau of Wuyishan City, Fujian Province, China. The tea samples were classified into four grades: Grand Prize (SA), First Prize (SB), Outstanding Award (SC), and Non-award (SD), in which the SA score was higher than 92.5, with an average score of 93.4; the SB score was higher than 86, with an average score of 88.38; the SC score was higher than 80, with an average score of 82.54; and the SD score was higher than 60, with an average score of 72.28. For each grade (SA, SB, SC, SD), three independent biological replicates were analyzed, resulting in a total of 12 samples. The four types of ‘Shuixian’ finished teas were all high-quality spring teas from the current year, sealed and kept in a refrigerator at 4 °C.

### 2.2. Electronic Nose Analysis

The volatile profiles of tea samples were analyzed using a PEN3 electronic nose system (Airsense Analytics GmbH, MV, Germany) using an optimized headspace sampling protocol adapted from Chen et al. [20]. The tea sample was removed from the refrigerator at 4 °C and left at room temperature for 1 h. Grind the sample using a grinder, then pass the ground tea powder through a 60-mesh (0.250 mm) sieve and collect the powder. For each measurement, 10 g of homogenized tea material was precisely weighed in a 250 mL borosilicate glass vessel. After adding 10 mL of deionized water maintained at 100 °C, the container was immediately sealed with gas-tight parafilm to establish a closed-headspace system. The samples underwent 30 min of static incubation at ambient temperature (25 ± 1 °C) to achieve vapor-phase equilibrium. Sensor array responses were recorded over a 90 s acquisition period, preceded by a 5 s purge cycle to eliminate atmospheric interference, with carrier gas flow maintained at 0.4 L min^−1^ throughout the detection phase. Sensor response data were autoscaled (mean-centered and unit-variance scaled) prior to principal coordinate analysis (PCoA) to eliminate magnitude-based bias. Variance stabilization ensured that all sensors contributed equally to multivariate modeling.

### 2.3. Extraction and Analysis of Tea Volatile Compounds

The volatile compounds in tea leaves were extracted and analyzed by headspace solid-phase microextraction (HS-SPME) combined with gas chromatography-mass spectrometry (GC-MS) [21]. The tea sample was removed from the refrigerator at 4 °C and left at room temperature for 1 h. Grind the sample using a grinder, then pass the ground tea powder through a 60-mesh (0.250 mm) sieve and collect the powder. Place 0.5 g of tea powder sample into a 20 mL headspace vial, add 10 mL of boiling water, seal the bottle, and place it in a constant temperature environment at 60 °C, stirring for 30 min to ensure the aroma components of the tea are fully released. Insert a 50/30 μm DVB/CAR/PDMS extraction head into the headspace of the sample vial and perform headspace extraction for 30 min. Then immediately insert the extraction needle into the GC-MS injection port and hold for 5 min. Experiments on each sample were triplicated.

GC conditions: DB-WAXetr column (60 m × 0.32 mm × 0.25 μm), carrier gas was high-purity helium (purity ≥ 99.999%), constant flow rate was 1.0 mL/min, inlet temperature was 250 °C, no shunt injection, and solvent delay was 3.5 min. Programmed temperature rise: 35 °C, hold for 5 min, ramp-up to 150 °C at 3 °C/min, ramp-up to 240 °C at 10 °C/min, hold for 2 min.

MS conditions: EI ionization mode; Ionization energy 70 eV; Ion source temperature 230 °C; Quadrupole temperature 150 °C; Emission current 34.6 μA; The quality scanning range was m/z 45~500 amu.

### 2.4. Qualitative and Quantitative Analysis of Tea Aroma Components

Volatile compounds were identified by comparing mass spectra from the National Institute of Standards and Technology Mass Spectrometry Library (NIST8.0), combining retention indexes, similarity matches, and relative abundance of fragment ions.

The n-alkane standard (C10-C20) was injected as an external standard under the same GC-MS conditions as the sample. Volatile compounds are calculated and reported based on external standard concentrations.

C10-C40 (even) n-alkanes standard (50 mg/L, 1 mL) were purchased from o2si Smart Solutions A LGC Standards Company (Charleston, SC, USA, official website: www.o2si.com, accessed on 1 March 2025). Solid Phase Microextraction (SPME) needle (50/30 µm, DVB/CAR/PDMS) was purchased from Supelco (Bellefonte, PA, USA).

### 2.5. Calculation of OAV of Teas and Analysis of Odor Characteristics

The threshold values for compounds were referenced in literature [22]. The OAV of each volatile compound (OAVi) was calculated as OAVi = Ci/Ti, where Ci was the content of compound i (µg/kg) and Ti was the threshold of compound i (µg/kg). Compounds with OAVi ≥ 1 were identified as odor-active compounds for further comparative analysis. The total OAV (OAVt) of tea was the sum of OAVi of odor-active compounds, i.e., OAVt = ∑OAVi. OAVt represented the aroma intensity of the tea.

According to the reference (Table S1) [23], the OAVi of compounds was divided into six odor characteristics, namely, woody, floral, burnt, green, fruity, and fatty. Then, the OAV values of each odor characteristic in tea were obtained.

### 2.6. Statistical Analysis

The data were preprocessed by mean centering and scaling prior to analysis. Principal component analysis (PCA), hierarchical cluster analysis (HCA, pheatmap 1.0.12), and orthogonal partial least squares discriminant analysis (OPLS-DA, package used for this was ropls and mixOmics) were performed by Chiplot (https://www.chiplot.online/, accessed on 14 April 2025). Given the sample size, model robustness was ensured through 200 permutation tests (OPLS-DA) and leave-one-out cross-validation (LOOCV). LOOCV achieved 91.7% classification accuracy (Table S5), indicating reliable model performance despite limited samples. Analysis of variance (ANOVA) was performed by SPSS (Version 22.0, IBM, Armonk, NY, USA). All data were presented as mean value ± SD. Differences between groups were reported to be significant at *p* < 0.05.

## 3. Results and Discussion

### 3.1. Electronic Nose Analysis of Wuyi Shuixian Tea in Different Grades

**Figure 1:**
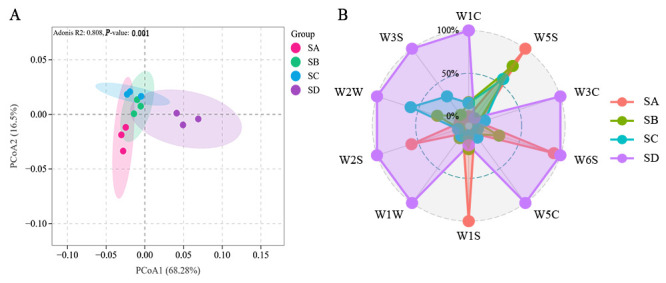
Electronic nose analysis of three grades of Wuyi Shuixian tea. (**A**) Principal Coordinates Analysis (PCoA) illustrating the differentiation between grades; (**B**) Radar chart depicting sensor responses (Normalize the data). SA: Grand Prize, SB: First Prize, SC: Outstanding Award, and SD: Non-award. W1C: Sensitive to aromatic compounds (benzenes), W5S: Sensitive to nitrogen oxides, W3C: Sensitive to ammonia water and aromatic components (amines), W6S: Selective for hydrogen (hydride), W5C: Sensitive to alkanes and aromatic components (short-chain alkanes), W1S: Sensitive to methane (methyl group), W1W: Sensitive to sulfides (inorganic sulfides), W2S: Sensitive to ethanol, W2W: Sensitive to organic sulfides, W3S: Sensitive to alkanes (long-chain alkanes).

The aroma analysis of different grades of Wuyi Shuixian using an electronic nose (Figure 1, Table S2) showed that Principal Coordinate Analysis (PCoA) could effectively distinguish the aroma differences between different grades of Wuyi Shuixian tea (*p* = 0.001, R^2^ = 0.808). PCoA1 accounted for 68.3% of the differences, while PCoA accounted for 16.5% (Figure 1A, Table S3). Further analysis of the response of each sensor (Figure 1B) showed that W5S (nitrogen oxides) increased with higher grades, while W2W (organic sulfides) decreased with increasing grade. This suggested that nitrogen oxides and organic sulfides were key substances contributing to the aroma differences between different grades of Wuyi Shuixian.

### 3.2. Analysis of Total Volatile Compounds in Different Grades of Wuyi Shuixian Tea

**Figure 2:**
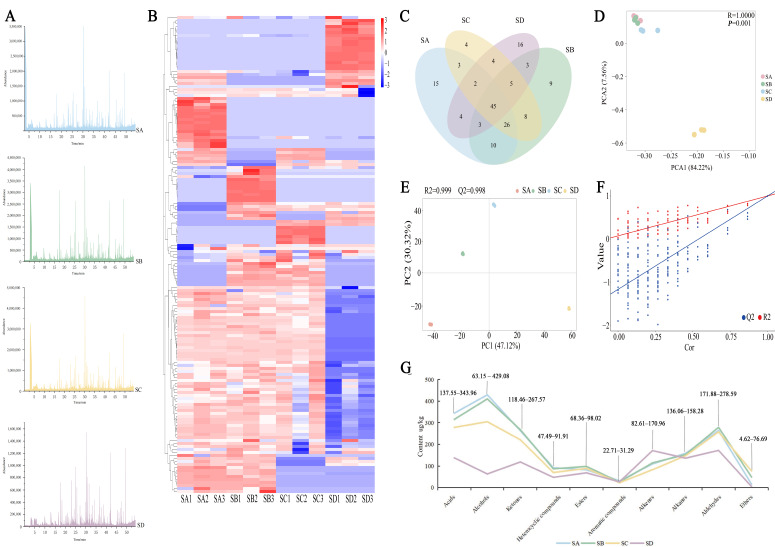
Analysis of total volatile compounds in different grades of Wuyi Shuixian tea (n = 3 per grade). (**A**) The total ions chromatogram, (**B**) Heat map of the volatile compounds, (**C**) VOCs Venn diagram; (**D**) Score scatter plot for PCA model (total); (**E**) PCA analysis of different grades of samples; (**F**) VOCs concentration line chart; (**G**) Content analysis of the ten types of volatile compounds. SA: Special prize; SB: first prize, SC: Excellent prize; SD: unnominated.

In order to study the aroma characteristics of different grades of Wuyi rock tea, HS-SPME and GC-MS were used to analyze and identify aroma compounds and their relative contents in four different grades of Shuixian samples. A total of 159 aroma components (Table S2) were detected in this experiment. According to the peak retention time, peak area value, relative content, and other related parameters of these aroma components, the ion spectra of different grades of Wuyi Shuixian tea were established (Figure 2A). As shown in the figure, the aroma components of the four grades of Shuixian samples were similar, but the total contents of each were different. The hierarchical clustering based on the visualization of heat maps was used to investigate the content differences of the four samples (Figure 2B). Combined with Figure 2C, it can be seen that only 45 compounds were common to Wuyi Shuixian tea of different grades, and there are 114 different compounds. The quantitative relationship of compounds of each grade was SA(108) ≈ SB(109) > SC(97) > SD(82), which may be one of the reasons for their difference. In addition, the Venn diagram results (Figure 2C) show that there are 10 common differentiated compounds between SA and SB: 2-(vinyloxy)ethanol, δ-hexalactone, phenylacetaldehyde, (+)-β-cedrene, 2,6-dimethylpyrazine, 4-tert-butylcyclohexanol, 2,3-pentanedione, 2-pentenal, 2-methylpentan-3-ol, and trans-nerolidol. These substances mainly exhibit floral, fruity, woody, and creamy aromas [24,25,26].

Principal Component Analysis (PCA) is the most widely used data dimension reduction algorithm. In this study, PCA analysis of aroma components of different grades of Shuixian was carried out (Figure 2D), and it was found that samples could be obviously separated by PCA pattern discriminant analysis based on VOC, with a certain position distance variation rule, in which PCA1 and PCA2 could be considered as the principal coordinate components that caused the difference between SD and the other three grades. Differences were explained by 84.2% and 7.6%, respectively, and the cumulative variance contribution rate reached 91.8%. The *p*-value of the total difference between different Shuixian grades determined in this study was 0.001, reaching the extremely significant level. PCA effectively segregated non-award SD from awarded grades (SA/SB/SC), but could not resolve finer distinctions within awarded tiers. The limited discrimination within awarded grades suggests complementary techniques (e.g., OPLS-DA) are needed for full grading.

Further classification of 159 aromatic components revealed that there were 7 acid compounds, 21 alcohol compounds, 27 ketone compounds, 10 heterocyclic compounds, 17 ester compounds, 11 aromatic compounds, 20 olefin compounds, 15 alkane compounds, 20 aldehyde compounds, and 11 other compounds, and PCA was carried out again based on this (Figure 2E–F, Table S5). The types of aroma components of different grades of samples were similar, but the total concentrations of all types were different (Figure 2G). The highest content of aroma components was represented by alcohols, followed by acids, aldehydes, and ketones. The contents of alcohols, acids, aldehydes, and ketones in SA were significantly higher than those in SD (*p* < 0.05). It is worth noting that among the 10 categories, only alkene content in SD was significantly higher than that of the other three grades (*p* < 0.05). Studies have shown that Shuixian, from better producing areas, accumulates more compounds that contribute a lot to tea aroma, mainly including alcohols, aldehydes, and ketones, while poor producing areas accumulate more hydrocarbons that contribute little to tea aroma [2,27]. Alcohol compounds usually have unique fragrances, such as floral, sweet, and woody, which have a good coordination effect on the aroma of tea. Among them, linalool, nerol, and α-terpineol can be biosynthesized through the terpenoid main chain [28]. In the process of oolong tea, endogenous glycosidase will hydrolyze the aromatic glycoside into alcohol. A total of 21 alcohols were identified in different grades of Shuixian tea, accounting for 24.0%, 22.8%, 19.6%, and 6.7% of the total content, respectively. Previous studies have found that linalool is one of the key aroma components for evaluating the quality of oolong tea [29]. The results of this experiment show that SA, SB, SC all have higher content of linalool, as shown by SA > SB > SC (*p* < 0.05). They accounted for 11.7%, 13.3%, and 8.2% of alcohol substances in the first three grades of Shuixian tea, respectively. However, no linalool was detected in SD. Aldehyde is an important contributor to the fragrance of flowers and fruits [30]. Furaldehyde and Hexanal, the main aldehyde compounds, exhibit aroma characteristics of semen armeniacae amarae and grass and apples, respectively. They were 55.9%, 51.4%, 48.1%, and 54.0% of the four grades of aldehydes. Further analysis of nitrogen oxides and sulfur compounds in tea leaves (Table S6) shows that the relationship trend between nitrogen oxide content and sulfur compounds is consistent across different grades of Wuyi Shuixian tea.

### 3.3. Screening of Volatile Components in Different Grades of Wuyi Shuixian Tea

**Figure 3:**
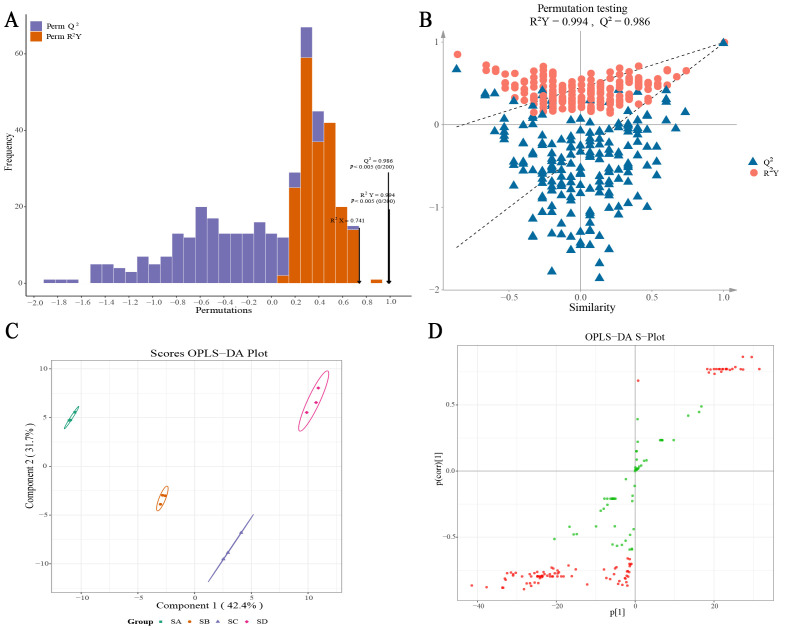
OPLS-DA analysis illustrating the differences in grades of Wuyi Shuixian tea (n = 3 per grade). (**A**) Test plot of OPLS-DA model for volatile components at different grades of Wuyi Shuixian tea; (**B**) The OPLS-DA model cross-validation results; (**C**) Scores OPLS-DA plot for analysis of within- and between-group differences in the different grades of Wuyi Shuixian tea; (**D**) OPLS-DA S-Plot screening of key volatile components differentiating various grades of Wuyi Shuixian tea.

To screen for different substances among different grades of Wuyi Shuixian tea, OPLS-DA analysis was performed on 159 aroma components. On this basis, this study further screened key differential volatile components using the OPLS-DA model. For the OPLS-DA model of different grades of Wuyi Shuixian tea, the goodness-of-fit (R^2^Y = 0.994, *p* < 0.005) and predictability (Q^2^ = 0.986, *p* < 0.005) of the model reached a significant level after 200 permutation tests (Figure 3A,B). LOOCV achieved 91.7% accuracy (11/12; Table S7). It can be seen that the OPLS-DA model constructed by different grades of Wuyi Shuixian tea met the requirements and could effectively distinguish between different samples, which could be utilized for further analysis. The results of the OPLS-DA plot analysis showed (Figure 3C) that different grades of Wuyi Shuixian tea could be effectively differentiated in different regions, with Component 1 showing a difference of 42.4% and Component 2 showing a difference of 31.7%. This indicates that there are significant differences between different grades of Wuyi Shuixian tea. Further S-Plot analysis of the constructed OPLS-DA model yielded variable importance in projection (VIP) values for different volatile compounds, identifying 99 key differential volatile components (Figure 3D).

### 3.4. Analysis of Key Compounds in Different Grades of Wuyi Shuixian Tea

**Figure 4:**
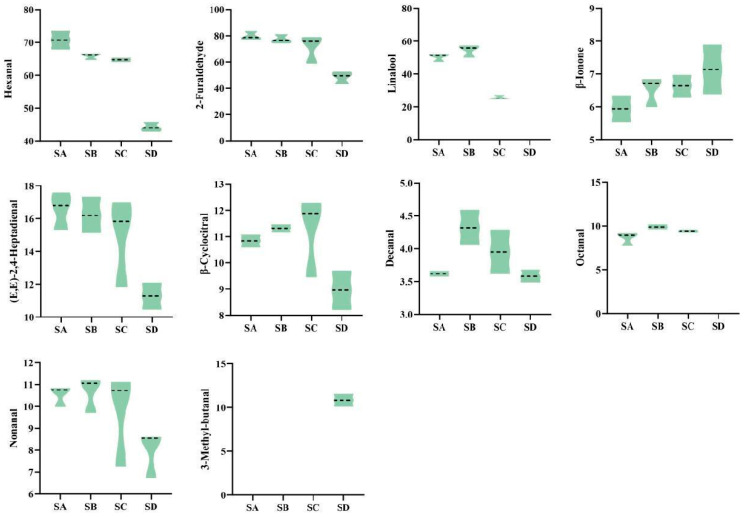
Volatile components with OAV > 1 and their contents in different grades of Wuyi Shuixian tea (ug/kg).

It is worthy of noting that not all volatile organic compounds (VOCs) contribute to the formation of tea aroma. The sensory effect of VOCs is not only influenced by concentration and odor characteristics but is closely related to threshold (Ti). Additionally, the types of aromas formed by compound combinations have significant differences in odor characteristics and proportions. OAV analysis is frequently used to assess the contribution of compounds to aroma; compounds with OAV > 1 are typically considered to have aroma activity and make a significant contribution to aroma characteristics. Quantitative analysis was performed on 99 volatile components with VIP values > 1, followed by calculation of the OAV values for different grades of Wuyi Shuixian tea based on reported thresholds and attribute descriptions of aroma components in literature. Finally, 10 compounds with OAV > 1 were identified (Figure 4), with SD having only 8 compounds, while the other three grades had 9 compounds each.

Zhu et al. [31] found that the main aroma components of Wuyi Shuixian tea are Hexanal, Linalool, β-Ionone, and Nerolidol. This study found that Furaldehyde (31.7%), Hexanal (28.1%), and Linalool (14.8%) accounted for a significant proportion of the total content of these 10 substances (Table S8), with Furaldehyde and Hexanal decreasing as the grade decreased, while Linalool, which has a floral aroma, first increased and then decreased, reaching its maximum value in SB. Notably, the content of β-Ionone, which imparts a violet aroma, showed a negative correlation with grade, indicated by SA < SB < SC < SD, and there was a highly significant difference between SA and SD (*p* < 0.05). Additionally, the trace apple-scented compound 3-Methyl-butanal was detected only in SD, with a content of 10.8 μg/kg, accounting for 7.6% of the total key compounds in SD.

When the OAV value is greater than 10, it is considered that the aroma component significantly contributes to the overall aroma of the tea [32]. In SA and SB, the main contributors are β-ionone, octanal, and linalool; in SC, β-ionone and octanal are the main contributors, while in SD, only one substance—β-ionone—plays a major role. This indicates that octanal and linalool are key components in SA, SB, and SC, and linalool content in the sample to some extent determines the award level of Wuyi Shuixian tea. Studies have shown that alcohols are particularly common aromatic compounds in tea, with diverse sources. Among them, terpene alcohols account for 51.26% of fresh spring tea leaves, and terpene alcohols are the primary aromatic substances in oolong tea, especially those with floral and fruity aromas such as linalool, oxidized linalool, and nerolidol [33,34,35,36]. Linalool and geraniol are considered important indicators for evaluating the quality of tea aroma, imparting a sweet floral scent to tea [37]. Therefore, it can be inferred that the presence of linalool is the main reason for the differences in grades in this study.

### 3.5. Correlation Analysis

**Figure 5:**
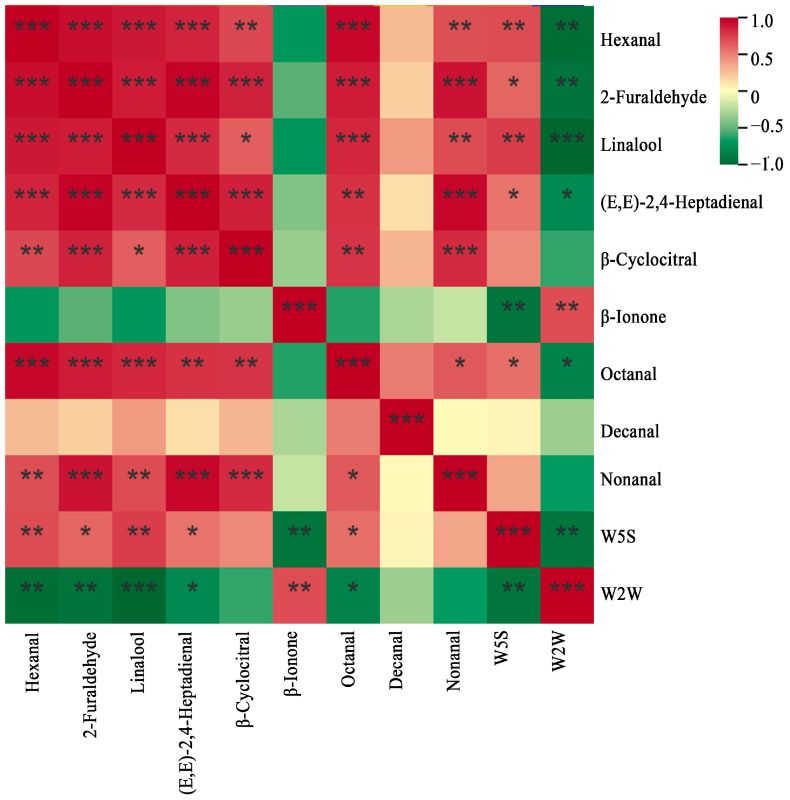
Analysis of the aromatic components and electronic nose correlation of different grades of Wuyi Shuixian tea. *: *p* < 0.05, **: *p* < 0.01, ***: *p* < 0.001.

Correlation analysis showed (Figure 5) that the W5S sensor of the electronic nose was significantly positively correlated with Hexanal, 2-Furaldehyde, Linalool, (E,E)-2,4-Heptadienal, and Octanal, and significantly negatively correlated with β-Ionone. The W2W sensor of the electronic nose was significantly and negatively correlated with Hexanal, 2-Furaldehyde, Linalool, (E,E)-2,4-Heptadienal, and Octanal, and significantly positively correlated with β-Ionone. Further elucidated that Octanal and linalool were the aromatic compounds present in high-quality Wuyi Shuixian tea.

### 3.6. Analysis of the Change of Scent Characteristics of Different Grades of Wuyi Shuixian Tea

**Figure 6:**
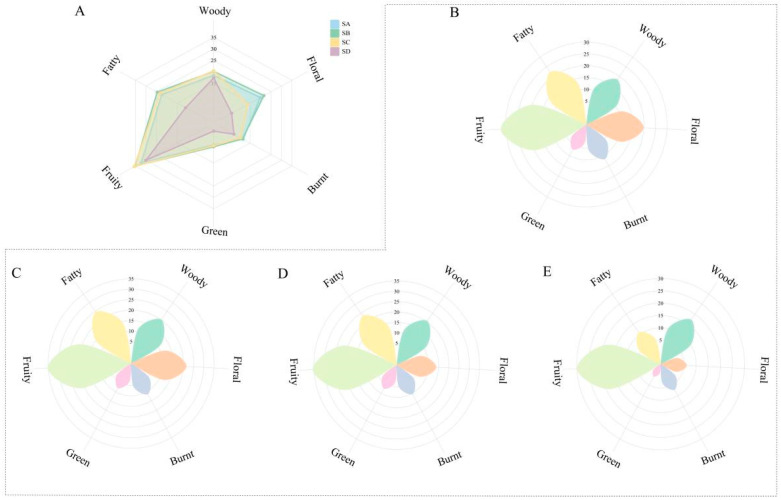
Radar chart of index of odor I(o) in different grades of Wuyi Shuixian tea. (**A**) Radar map of odor contribution of key compounds; (**B**) Radar map of odor contribution of SA; (**C**) Radar map of odor contribution of SB; (**D**) Radar map of odor contribution of SC; (**E**) Radar map of odor contribution of SD.

The aroma characteristics of compounds in tea leaves are mainly classified into six types: woody, floral, burnt, green, fruity, and fatty [38]. This study further categorized these 10 key compounds based on their aroma characteristics and combined them with OAV values to calculate OAV and OAVt for these 10 compounds across the six aroma categories. The OAV values for these six aroma characteristics showed different distributions among different levels (Tables S9–S12). Regarding the variation in odor characteristics across four levels, woody, green, and fruity aroma traits showed a negative correlation in the three award-winning levels (Figure 6A), indicated by SA < SB < SC. Additionally, the OAV values of SD were all lower than those of the other three levels; burnt and fatty had no significant differences among SA, SB, and SC but decreased with the descending level, i.e., SA ≈ SB ≈ SC > SD; interestingly, this study found that floral had no significant difference between SA and SB, with SA (19.9) < SB (21.6), showing highly significant differences from SC and SD.

Further analysis of the aroma composition characteristics of different grades of Wuyi Shuixian tea revealed that the OAVt (111.7) of SA consists of 32.2 fruity, 22.4 fatty, 19.9 floral, 18.4 woody, 11.7 burnt, and 7.1 green components, indicating that fruity is the predominant component in SA, with fatty, floral, and woody components evenly distributed, supplemented by burnt and green, ultimately forming the aroma type of SA (Figure 6B). The OAVt (120.8) of SB consists of 35.2 fruity, 24.6 fatty, 21.6 floral, 20.2 woody, 11.3 burnt, and 7.9 green components, similar to the overall composition of SA (Figure 6C). The OAVt of SC is 111.2, consisting of 35.6 fruity, 23.4 fatty, 20.4 woody, 13.9 floral, 10.4 burnt, and 7.4 green components, with fruity being the main aromatic substance, fatty and floral components evenly distributed, and finally supplemented by burnt and green (Figure 6D). The OAVt (72.8) of SD consists of 30.0 fruity, 17.3 woody, 11 fatty, 7.2 burnt, 6.2 floral, and 1.1 green components, indicating that although SD is also predominantly fruity, it has woody and fatty as secondary aromas, followed by floral, burnt, and green (Figure 6E).

Compared to Wuyi Rougui tea and Ruixiang tea, Wuyi Shuixian tea exhibits higher expression levels of β-prunasin glycosidase, indicating a more intense natural floral aroma [39,40]. Wuyi Shuixian tea is known for its rich and lingering fragrance, reminiscent of orchids but surpassing them, with the most esteemed varieties being those with fresh and distant aromas. Research has found that Wuyi Shuixian tea mainly consists of trans-neryl acetate, (E,E)-2,4-heptadienal, linalool, and its oxides, where both trans-neryl acetate and linalool are key contributors to the floral scent [41]. In this study, the floral aroma of grades from SA and SB was more pronounced than other aromatic characteristics, resulting in significant differences between SA, SB, and other grades. During the Oolong tea evaluation process, the final score is derived from a weighted average of aroma (30%), taste (35%), appearance (20%), liquor color (5%), and leaf base (10%). This indicates that aroma is not the only factor influencing the final score, as taste also plays a substantial role. Consequently, despite SB having a floral content similar to SA, its grade is lower than SA.

## 4. Conclusions

This study systematically investigated the aroma characteristics and key odor-active compounds of Wuyi Shuixian tea across four grades (SA, SB, SC, SD) using HS-SPME-GC-MS combined with multivariate statistical analysis and the OAV-splitting method. A total of 159 volatile compounds were identified, with similar compound types but distinct concentration gradients between grades. OAV analysis revealed that β-ionone (fruity), octanal (fatty), and linalool (floral) served as core aroma-active compounds, contributing significantly to sensory perception. Notably, linalool, a critical floral marker, exhibited a concentration gradient (SA > SB > SC) and was undetected in SD, suggesting linalool as a potential determinant.

Multivariate analysis further demonstrated that SA, SB, and SC were characterized by balanced fruity, floral, fatty, and woody aromas, while SD lacked floral traits and instead displayed burnt notes. The pronounced disparity in floral characteristics between awarded (SA, SB, SC) and non-awarded (SD) grades underscored the importance of linalool in quality evaluation. Additionally, electronic nose data corroborated these findings, showing strong correlations between sensor responses (W5S and W2W) and key compounds like hexanal and β-ionone.

These results provide a molecular basis for understanding the relationship between aroma profiles and tea grades, providing actionable insights for refining processing techniques and enhancing quality control standards. Future studies should focus on dynamic changes in aroma compounds during processing and the influence of terroir on VOC biosynthesis, further bridging sensory attributes with chemical fingerprints to advance precision in tea industry practices.

## Data Availability

The original contributions presented in this study are included in the article/Supplementary Materials. Further inquiries can be directed to the corresponding authors.

## Supplementary Materials

The following supporting information can be downloaded at: https://www.mdpi.com/article/10.3390/foods14132169/s1, Table S1: The threshold value and odor characteristics of 10 key compounds; Table S2: Aromatic components of different grades of Wuyi Shuixian tea; Table S3: Analysis of raw data for different grades of Wuyi Shuixian using an electronic nose; Table S4: Electronic nose data component matrix; Table S5: Classification of aroma grades for Wuyi Shuixian tea; Table S6: The content of nitrogen oxides and sulfur compounds in the aroma components of different grades of Wuyi Shuixian tea (μg/kg); Table S7: OPLS-DA classification confusion matrix; Table S8: The content of compounds with OAV > 1 in different grades of Wuyi Shuixian tea; Table S9: The content and OAV-splitting of OAV > 1 compounds in SA; Table S10: The content and OAV-splitting of OAV > 1 compounds in SB; Table S11: The content and OAV-splitting of OAV > 1 compounds in SC; Table S12: The content and OAV-splitting of OAV > 1 compounds in SD.
