# Supplementary material for: Aroma-Driven Differentiation of Wuyi Shuixian Tea Grades: The Pivotal Role of Linalool Revealed by OAV and Multivariate Analysis
Source: Foods. 2025 Jun 21;14(13):2169. doi: 10.3390/foods14132169 (PMC12249095; doi:10.3390/foods14132169)
Supplement: Supplementary file 1 [file foods-14-02169-s001.zip › foods-3666402-supplementary.pdf]

Table S1 The threshold value and odor characteristics of 10 key compounds

No.	Compound	CAS	Ti (ug/kg)	Split of odor characteristic (%)*					
				Woody	Floral	Burnt	Green	Fruity	Fatty
1	Hexanal	66-25-1	30	10	0	0	10	20	60
2	Furaldehyde	98-01-1	8	0	0	100	0	0	0
3	(*E*,*E*)-2,4-Heptadienal	4313-03-5	3.5	0	0	36	16	0	48
4	β-Ionone	14901-07-6	0.104	30	10	0	0	50	10
5	Linalool	78-70-6	3.8	0	100	0	0	0	0
6	β-Cyclocitral	432-25-7	3	30	10	0	0	50	10
7	Octanal	124-13-0	0.52	0	0	0	35	5	60
8	Decanal	112-31-2	3.02	0	0	0	0	20	80
9	Nonanal	124-19-6	45	0	20	0	10	10	60
10	3-Methyl-butanal	590-86-3	0.008	0	0	0	15	45	40

Table S2 Analysis of raw data for different grades of Wuyi Shuixian using an electronic nose

Ind ex	SA-1	SA-2	SA-3	SB-1	SB-2	SB-3	SC-1	SC-2	SC-3	SD-1	SD-2	SD-3
W1	2426	2417	2427	2424	2430	2425	2427	2423	2428	2453	2457	2451
C	8	3	8	4	3	5	7	4	6	9	5	0
W5	2477	2460	2468	2457	2454	2456	2447	2441	2444	2439	2414	2426
S	6100	6786	9674	3842	4872	0514	0516	5040	8684	0254	5380	0902
W3	2475	2475	2478	2478	2475	2474	2479	2475	2478	2576	2510	2506
C	4	2	7	2	5	5	5	5	6	1	5	3
W6	1093	1106	1081	1083	1079	1065	1066	1060	1062	1095	1088	1104
S	5	8	4	7	4	9	1	2	2	0	9	1
W5	2918	2923	2923	2925	2917	2922	2924	2921	2924	2964	2964	2960
C	9	2	0	8	9	0	4	3	4	7	9	7
W1	7530	7610	7569	7406	7356	7409	7377	7378	7380	7352	7388	7382
S	8	9	2	3	6	2	8	3	7	5	1	8
W1	9287	9382	9429	9453	9575	9579	9570	9422	9359	9930	9885	9983
W	52	05	34	17	51	67	27	08	04	62	12	60
W2	1582	1528	1558	1566	1565	1558	1562	1550	1549	1582	1585	1582
S	311	870	619	135	199	793	775	381	209	030	714	080
W2	2057	2058	2057	2080	2059	2061	2073	2075	2074	2088	2086	2089
W	7434	8452	2016	8618	3022	0710	6104	2820	9666	9486	6654	6012
W3	7115	7145	7116	7133	7149	7142	7178	7172	7177	7538	7257	7267
S	2	4	9	4	3	8	4	9	4	8	4	5

Table S3 Electronic nose data component matrix

	Principal Component 1	Principal Component 2
W1C	0.957	0.087

W5S	-0.808	0.348
W3C	0.869	0.222
W6S	0.334	0.88
W5C	0.962	0.187
W1S	-0.583	0.747
W1W	0.934	0.031
W2S	0.699	0.086
W2W	0.864	-0.248
W3S	0.864	0.106

Table S4 Aromatic components of different grades of Wuyi Shuixian tea

	Retain time	Compounds	CAS	SA	SB	SC	SD
1	8.674	Pentanal	000110-62-3	19.858	30.4676	36.7539	4.5511
2	12.848	Hexanal	000066-25-1	70.7344	65.8287	64.7065	44.2112
3	13.511	Undecane	001120-21-4	20.6508	2.953	6.2174	2.7121
4	18.329	Dodecane	000112-40-3	35.4279	34.92	39.9269	40.2403
5	20.032	Furan, 2-pentyl-	003777-69-3	9.7742	9.0823	7.0458	12.4681
6	21.615	Methyl-pyrazine	000109-08-0	24.6631	25.011	22.9955	15.7978
7	23.12	Tridecane	000629-50-5	52.7954	60.4107	70.9261	55.156
8	23.815	Cyclohexanone, 2,2,6-trimethyl-	002408-37-9	8.7153	13.997	10.6704	10.4004
9	25.065	6-methyl-5-Hepten-2-one	000110-93-0	38.4339	46.4383	38.8121	41.9792
10	27.108	3-Hexen-1-ol, (Z)-	000928-96-1	32.7672	31.4924	31.4924	21.7386
11	27.579	Nonanal	000124-19-6	10.5269	10.6567	9.6991	7.9635
12	27.688	Tetradecane	000629-59-4	14.1294	17.1527	13.0808	14.4659
13	30.147	Acetic acid	000064-19-7	271.2507	253.5714	219.7209	111.9585
14	30.848	Furfural	000098-01-1	79.8064	77.3762	71.2424	48.5152
15	31.597	2-Propenoic acid, 2-ethylhexyl ester	000103-11-7	30.0967	37.3748	38.0749	30.4431
16	31.802	2-Ethyl-1-hexanol	000104-76-7	8.6522	10.0843	10.0418	6.5252
17	32.026	2,4-Heptadienal, (E,E)-	004313-03-5	16.5548	16.2249	14.8833	11.2789
18	32.592	Ethanone, 1-(2-furanyl)-	001192-62-7	7.5546	6.9491	7.3343	5.6827
19	33.125	3,5-Octadien-2-one	038284-27-4	9.4525	8.942	7.0278	4.8806
20	33.289	Benzaldehyde	000100-52-7	20.5228	20.4242	19.7547	16.6611
21	33.923	Propanoic acid	000079-09-4	16.2525	14.9972	14.0023	3.7619
22	34.738	1H-3a,7-Methanoazulene, 2,3,4,7,8,8a-hexahydro-3,6,8,8-tetramethyl-, [3R-(3.alpha.,3a.beta.,7.beta.,8a.alpha.)]-	000469-61-4	4.1033	4.0086	3.873	2.99
23	35.452	2-Furancarboxaldehyde, 5-methyl-	000620-02-0	8.6688	7.6666	7.9892	6.3316

24	36.195	Hexadecane	000544-76-3	6.1962	6.3921	4.5395	8.1121
25	37.131	1-Cyclohexene-1-carboxaldehyde, 2,6,6-trimethyl-	000432-25-7	10.836	11.3188	11.2026	8.9581
26	37.603	Cyclohexanone, 4-(1,1-dimethylethyl)-	000098-53-3	31.9002	30.1405	29.0121	17.6024
27	38.627	Hexanoic acid, 3-hexenyl ester, (Z)-	031501-11-8	21.9764	25.0073	23.689	16.0779
28	38.932	2-Furanmethanol	000098-00-0	16.1961	15.0081	15.0182	9.1382
29	39.844	Naphthalene, 1,2,3,4-tetrahydro-1,1,6-trimethyl-	000475-03-6	3.9542	4.0362	4.1449	3.2451
30	40.456	2(3H)-Furanone, 5-ethyldihydro-	000695-06-7	18.2269	16.4309	15.5793	8.6204
31	41.639	2,5-Furandione, 3,4-dimethyl-	000766-39-2	3.4631	3.598	3.5085	3.2034
32	42.152	.alpha.-Farnesene	000502-61-4	73.2502	74.2808	61.0698	70.9253
33	42.527	2(5H)-Furanone	000497-23-4	4.4824	4.531	4.1793	3.2129
34	45.391	Hexanoic acid	000142-62-1	28.4685	33.4806	29.3019	9.5863
35	46.23	Benzyl Alcohol	000100-51-6	11.6038	10.8995	10.882	7.0633
36	46.351	2,5-Pyrrolidinedione, 1-ethyl-	002314-78-5	6.0968	6.1963	6.371	7.0863
37	47.022	Phenylethyl Alcohol	000060-12-8	13.1998	12.9373	12.8856	8.5383
38	47.548	3-Buten-2-one, 4-(2,6,6-trimethyl-1-cyclohexen-1-yl)-, (E)-	000079-77-6	5.9398	6.5201	6.6371	7.1409
39	47.644	2-Cyclopenten-1-one, 3-methyl-2-(2-pentenyl)-, (Z)-	000488-10-8	4.3713	4.5651	4.7505	2.7351
40	47.928	3-Hexenoic acid, (E)-	001577-18-0	10.0953	10.6711	9.7418	3.0656
41	48.273	Ethanone, 1-(1H-pyrrol-2-yl)-	001072-83-9	13.4728	12.0134	11.1515	5.0007
42	49.233	1H-Pyrrole-2-carboxaldehyde	001003-29-8	1.2364	0.7072	0.7168	0.0705
43	52.611	(Z)-tetrahydro-6-(2-pentenyl)-2H-pyran-2-one	025524-95-2	9.2484	7.7449	7.7962	2.5609
44	53.765	5,6,7,7a-tetrahydro-4,4,7a-trimethyl-2(4H)-Benzofuranone	015356-74-8	3.3598	4.3276	3.9684	1.971
45	4.752	Acetone	000067-64-1	27.0925	52.3145	24.3674	0
46	10.661	Eicosane	000112-95-8	3.8237	3.9459	0	3.2626
47	15.507	Benzene, 1,3-dimethyl-	000108-38-3	7.5194	3.9525	3.2276	0
48	16.601	1-Penten-3-ol	000616-25-1	43.7697	46.3127	46.2877	0
49	17.888	Hexanoic acid, methyl ester	000106-70-7	5.1222	5.6736	0	3.8456
50	19.035	1,3-Diazine	000289-95-2	6.3387	5.1952	4.5022	0
51	20.908	1-Pentanol	000071-41-0	34.3948	34.4106	28.1718	0
52	21.422	3-Hexenoic acid, methyl ester, (Z)-	013894-62-7	3.5925	4.5636	4.1559	0
53	22.24	Benzene, 1,2,3-trimethyl-	000526-73-8	0	10.1239	7.8362	8.8552
54	22.751	Octanal	000124-13-0	8.6171	9.8873	9.3834	0
55	23.313	2-Propanone, 1-hydroxy-	000116-09-6	54.3985	35.0246	25.9442	0
56	24.268	2-Penten-1-ol, (Z)-	001576-95-0	35.6236	37.1237	32.4068	0
57	24.818	Pyrazine, ethyl-	013925-00-3	13.4056	13.0091	12.2082	0

58	26.721	1-Hydroxy-2-butanone	005077-67-8	4.1086	3.4625	3.8158	0
59	27.845	4,5-dihydro-5,5-dimethyl-4-isopropylidene-1H-Pyrazole	106251-09-6	4.9786	6.3803	4.3905	0
60	28.171	2-Hexen-1-ol, (E)-	000928-95-0	12.1068	7.8284	9.998	0
61	29.301	Benzene, 1,2,3,4-tetramethyl-	000488-23-3	0	5.45	2.2935	3.0413
62	29.361	Benzene, 1,2,4,5-tetramethyl-	000095-93-2	7.4421	0	4.5976	3.6117
63	31.089	cis-3-Hexenyl isovalerate	035154-45-1	5.8099	6.5568	6.3863	0
64	32.183	Decanal	000112-31-2	3.6208	4.3246	3.9502	3.5847
65	34.279	3,7-dimethyl-1,6-Octadien-3-ol	000078-70-6	50.2227	54.4219	24.9263	0
66	34.63	1-Octanol	000111-87-5	6.1701	5.3136	5.1378	0
67	36.859	1,5,7-Octatrien-3-ol, 3,7-dimethyl-	029957-43-5	55.9882	51.8432	57.09	0
68	38.116	1,3-Cyclohexadiene-1-carboxaldehyde, 2,6,6-trimethyl-	000116-26-7	7.0778	5.1421	4.7124	0
69	43.24	Methyl salicylate	000119-36-8	4.1742	4.7029	0	3.1098
70	45.119	2-Azabicyclo[2.2.1]heptane	1000338-38-9	0	5.8824	5.0429	3.7033
71	46.164	Propanoic acid, 2-methyl-, 1-(1,1-dimethylethyl)-2-methyl-1,3-propanediyl ester	074381-40-1	2.9317	2.9737	2.8779	0
72	47.221	1,4-Butanediol	000110-63-4	2.324	2.3436	2.4546	0
73	47.819	Heptanoic acid	000111-14-8	2.4156	2.6638	2.2679	0
74	48.593	3-Buten-2-one, 4-(2,2,6-trimethyl-7-oxabicyclo[4.1.0]hept-1-yl)-	023267-57-4	0.8323	0.9829	0.9891	0
75	51.608	Caprolactam	000105-60-2	1.2686	2.065	1.9996	0
76	52.834	4H-Pyran-4-one, 2,3-dihydro-3,5-dihydroxy-6-methyl-	028564-83-2	3.9164	4.9283	5.6984	0
77	48.877	Phenol	000108-95-2	0.1115	0.13	0.0858	0
78	3.919	Ethylene oxide	000075-21-8	1.7416	0	0	0
79	3.949	Acetaldehyde	000075-07-0	0	2.5716	0	0
80	7.87	Heptane, 2,2,4,6,6-pentamethyl-	013475-82-6	7.5783	0	0	0
81	8.813	Propane, 2-(ethenyloxy)-	000926-65-8	5.9832	0	0	0
82	8.828	3-Methyl-butanal	000590-86-3	0	0	0	8.3065
83	10.164	7-methyl-7H-Dibenzo[b,g]carbazole	003557-49-1	0	0	0	2.97
84	10.705	3-Ethyl-3-methylheptane	017302-01-1	0	0	0	2.7123
85	10.994	Toluene	000108-88-3	0.9465	0	0	0
86	17.743	Heptanal	000111-71-7	4.4356	0	0	0
87	18.151	Limonene	000138-86-3	0	0	0	6.0196
88	19.334	2-Hexenal	000505-57-7	0	0	0	8.3289
89	19.579	Benzene, 1-ethyl-3-methyl-	000620-14-4	6.0985	0	0	0
90	20.159	Bicyclo[3.1.1]hept-2-ene,	002437-95-8	2.1691	0	0	0

		2,6,6-trimethyl-, (.+/-.)-					
91	20.6	Octane, 5-ethyl-2-methyl-	062016-18-6	17.6823	0	0	0
92	21.259	Bicyclo[4.2.0]octa-1,3,5-triene	000694-87-1	7.0136	0	0	0
93	21.439	3-Hexenoic acid, methyl ester	002396-78-3	0	0	0	3.8459
94	23.893	2-Penten-1-ol, (E)-	001576-96-1	7.1556	0	0	0
95	25.192	1-Octadecene	000112-88-9	0	0	0	5.6174
96	26.153	10-Methyl-eicosane	054833-23-7	0	3.6578	0	0
97	26.22	1-Iodo-2-methylundecane	073105-67-6	0	0	3.7513	0
98	26.286	Benzene, 1-methyl-2-(1-methylethyl)-	000527-84-4	2.9851	0	0	0
99	26.645	2,4,6-Octatriene, 2,6-dimethyl-, (E,Z)-	007216-56-0	0	0	0	2.2877
100	27.325	Bicyclo[2.2.1]hept-2-ene, 1,7,7-trimethyl-	000464-17-5	0	11.8553	0	0
101	27.38	Cyclohexene, 1-methyl-4-(1-methylethylidene)-	000586-62-9	10.2692	0	0	0
102	28.334	Propanenitrile	000107-12-0	2.8484	0	0	0
103	28.461	1,3-Hexadiene, 3-ethyl-2-methyl-	061142-36-7	0	1.8596	0	0
104	28.576	Butanoic acid, octyl ester	000110-39-4	3.3427	0	0	0
105	28.588	Butanoic acid, hexyl ester	002639-63-6	0	0	3.4539	0
106	29.974	Trichloroacetic acid, 2-ethylhexyl ester	016397-79-8	0	0	0	3.6635
107	30.576	cis-3-Hexenyl iso-butyrate	041519-23-7	0	5.7011	0	0
108	30.582	Butanoic acid, 3-hexenyl ester, (Z)-	016491-36-4	0	0	5.1069	0
109	31.228	Succinic acid, decyl octyl ester	1000324-96-3	5.2442	0	0	0
110	31.887	Octadecane, 3-ethyl-5-(2-ethylbutyl)-	055282-12-7	0	3.6612	0	0
111	34.286	3-Carene	013466-78-9	0	0	0	17.8593
112	36.862	E,E-2,6-Dimethyl-1,3,5,7-octatetraene	000460-01-5	0	0	0	43.6916
113	38.033	Benzenamine, 4-methoxy-2-methyl-	000102-50-1	0	0	0	4.622
114	38.044	Benzenamine, 4-methoxy-N-methyl-	005961-59-1	0	0	5.3785	0
115	38.444	Acetophenone	000098-86-2	0	0	0	4.6211
116	39.155	Silver hexanoate	032461-90-8	0	22.6252	0	0
117	40.062	5,5-Dimethyl-3-oxo-1-cyclohexene-1-carboxaldehyde	056621-35-3	0	0	0	3.1201
118	40.164	3,7,7-Trimethyl-bicyclo[4.1.0]hept-2-ene	000554-61-0	0	3.2737	0	0
119	40.624	2-ethyl-1,2,3-Butanoic acid, propanetriyl ester	056554-54-2	0	0	5.0107	0

120	41.3	1,3,6,10-Dodecatetraene, 3,7,11-trimethyl-, (Z,E)-	026560-14-5	6.2268	0	0	0
121	41.965	Cyclohexanol, 4-(1,1-dimethylethyl)-, trans-	021862-63-5	24.519	20.0585	0	0
122	41.968	Cyclohexene, 4-(1,1-dimethylethyl)-	002228-98-0	0	0	0	14.1619
123	42.721	6-ethenyltetrahydro-2,2,6-trimeth yl-2H-Pyran-3-ol	014049-11-7	0	0	0	5.5689
124	44.859	4-Butyl-indan-5-ol	1000194-67-3	0	2.8119	0	0
125	45.65	5,9-Undecadien-2-one, 6,10-dimethyl-, (E)-	003796-70-1	0	4.1449	0	0
126	46.167	Pentanoic acid, 2,2,4-trimethyl-3-carboxyisoprop yl, isobutyl ester	1000140-77-5	0	0	0	1.7822
127	49.354	2(3H)-Furanone, dihydro-3-hydroxy-4,4-dimethyl-, (.+/-.)-	000079-50-5	1.5061	0	0	0
128	49.421	1,6,10-Dodecatrien-3-ol, 3,7,11-trimethyl-, [S-(Z)]-	000142-50-7	0	0	51.5926	0
129	4.281	Propanal	000123-38-6	0	3.7517	2.0568	0
130	5.055	Acetic acid, hydrazide	001068-57-1	12.4171	0	0	9.1816
131	12.057	2,3-Pentanedione	000600-14-6	4.4911	4.4271	0	0
132	15.03	2-Pentenal, (E)-	001576-87-0	11.4776	8.7704	0	0
133	15.126	3-Penten-2-one, 4-methyl-	000141-79-7	16.156	0	11.0882	0
134	15.489	o-Xylene	000095-47-6	0	2.3468	2.1217	0
135	15.555	p-Xylene	000106-42-3	0	3.6679	2.5525	0
136	15.815	1-Butanol	000071-36-3	4.2317	0	0	10.1438
137	15.894	1-Hexanol	000111-27-3	10.5759	0	17.7789	0
138	16.438	3-Pentanol, 2-methyl-	000565-67-3	2.9211	4.7914	0	0
139	18.117	D-Limonene	005989-27-5	0	6.0091	4.3786	0
140	19.241	2-Hexenal, (E)-	006728-26-3	0	13.8068	12.5927	0
141	20.473	Benzene, 1,3,5-trimethyl-	000108-67-8	6.1868	0	0	11.121
142	23.458	Nonadecane	000629-92-5	0	10.8506	0	5.6962
143	24.522	Pyrazine, 2,6-dimethyl-	000108-50-9	11.7196	9.1337	0	0
144	27.839	2-Cyclohexen-1-one, 3,5,5-trimethyl-	000078-59-1	0	5.2602	0	2.9375
145	28.497	(Z)-3-ethyl-2-methyl-1,3-Hexadie ne	074752-97-9	2.3435	2.1074	0	0.8626
146	28.999	Butyl 2-methylbutanoate	015706-73-7	0	5.4719	0	5.5924
147	30.666	(R,S)-5-Ethyl-6-methyl-3E-hepten -2-one	057283-79-1	0	0	5.9682	5.0097
148	34.884	1H-3a,7-Methanoazulene, octahydro-3,8,8-trimethyl-6-meth	000546-28-1	2.8519	3.1133	0	0

		ylene-, [3R-(3.alpha.,3a.beta.,7.beta.,8a.alpha.)]-					
149	36.267	n-Nonylcyclohexane	002883-02-5	0	2.6317	3.5864	0
150	36.328	2,6-dimethyl-cyclohexanol	005337-72-4	0	18.5134	17.7237	0
151	38.255	Benzeneacetaldehyde	000122-78-1	4.4382	2.7594	0	0
152	40.067	2,6,6-Trimethyl-2-cyclohexene-1,4-dione	001125-21-9	3.9429	0	4.3323	0
153	41.3	Bicyclo[3.1.1]hept-2-ene, 2,6-dimethyl-6-(4-methyl-3-pentenyl)-	017699-05-7	0	6.713	5.3531	0
154	43.064	Benzene, 1-(1,5-dimethyl-4-hexenyl)-4-methyl-	000644-30-4	0	0	3.7962	3.2998
155	43.789	2H-Pyran-2-one, tetrahydro-6-methyl-	000823-22-3	2.8212	2.9576	0	0
156	48.103	2-Hexenoic acid	001191-04-4	3.0617	0	3.4017	0
157	48.78	4-(2,6,6-Trimethylcyclohexa-1,3-dienyl)but-3-en-2-one	001203-08-3	0	0.6342	0.6171	0
158	49.427	1,6,10-Dodecatrien-3-ol, 3,7,11-trimethyl-, (E)-	040716-66-3	45.1755	53.5496	0	0
159	53.07	Ethanol, 2-(vinyloxy)-	000764-48-7	0.765	2.4989	0	0

Table S5 Classification of aroma grades for Wuyi Shuixian tea

Index	SA	SB	SC	SD
Acids	343.96±7.61a	315.38±9.91a	278.44±10.35b	137.55±6.04c
Heterocyclic compound	91.91±2.99a	86.77±0.35a	69.63±6.92b	47.49±3.38c
Aromatic compounds	31.29±0.86a	25.67±2.04ab	22.71±2.69b	26.63±1.64ab
Ethers	15.46±6.30c	48.82±0.93b	76.69±2.85a	4.62±0.17c
Alkanes	158.28±5.16a	152.46±6.15a	147.07±13.09a	136.06±9.79a
Esters	82.29±7.99bc	98.03±2.04a	88.76±1.84ab	68.36±2.96c
Aldehydes	266.94±13.64a	282.91±5.42a	269.64±9.01a	171.88±8.07b
Alkenes	109.33±13.13b	114.14±7.08b	82.62±8.71c	170.96±3.60a
Alcohols	429.07±17.71a	410.00±6.96a	304.57±24.26b	63.15±10.85c

Ketones	262.96±8.81ab	267.57±18.10a	221.13±19.02b	119.96±4.90c

Note: Different lowercase letters on the same line indicate significant data differences (P < 0.05).

Table S6 The content of nitrogen oxides and sulfur compounds in the aroma components of different grades of Wuyi Shuixian tea (μ g/kg)

	Compounds	SA	SB	SC	SD
Nitrogen oxides	Heterocyclic compound	24.66	25.01	23	15.8
	2-Ethylpyrazine	13.41	13.01	12.21	
	2,6-Dimethylpyrazine	11.72	9.13	0	0
	1-(1H-Pyrrol-2-yl)ethanone	13.47	12.01	11.15	5
	Pyrrole-2-carboxaldehyde	1.24	0.71	0.72	0.07
	Propionitrile	2.85	0	0	0
Sulfur compounds	N-Ethylsuccinimide	6.1	6.2	6.37	7.09

Table S7 OPLS-DA classification confusion matrix

True level	Forecast SA	Forecast SB	Forecast SC	Forecast SD	Accuracy
SA	3	0	0	0	100%
SB	0	3	0	0	100%
SC	0	1	2	0	66.70%
SD	0	0	0	3	100%
Total					91.70%

Table S8 The content of compounds with OAV>1 in different grades of Wuyi Shuixian tea

	Compound	SA	SB	SC	SD	Total	%
1	Hexanal	70.73±2.78a	65.83±1.01a	64.71±0.59a	44.21±1.41b	245.48	28.13
2	Furaldehyde	79.81±3.28a	77.38±3.35a	71.24±3.69a	48.52±4.62b	276.95	31.74
3	Linalool	50.22±2.38b	54.42±3.76a	24.93±0.10c	-	129.57	14.85
4	(*E*,*E*)-2,4-Heptadienal	16.55±1.16a	16.22±1.09a	14.88±2.68a	11.28±0.8b	58.93	6.75
5	β-Cyclocitral	10.84±0.24a	11.32±0.15a	11.21±1.52a	8.96±0.74b	42.33	4.85
6	β-Ionone	5.94±0.40b	6.52±0.46ab	6.64±0.34ab	7.14±0.75a	26.24	3.01
7	Octanal	8.62±0.76b	9.89±0.28a	9.38±0.11ab	-	27.89	3.21
8	Decanal	3.62±0.04b	4.32±0.27a	3.95±0.33ab	3.58±0.10b	15.47	1.77
9	Nonanal	10.53±0.47a	10.66±0.82a	9.7±2.12ab	7.96±1.06b	38.85	4.45
10	3-Methyl-butanal	-	-	-	10.84±0.7	10.84	1.24

Note: "-" indicates not detected in the sample. Significant differences are indicated between different letters.

Table S9 The content and OAV-splitting of OAV>1 compounds in SA.

Compound	Ci ug/kg	OAVi	%	Split of odor characteristic (%)					
				Woody	Floral	Burnt	Green	Fruity	Fatty
Hexanal	70.73±2.78	2.26	2.01	0.23	0	0	0.23	0.45	1.35
Furaldehyde	79.81±3.28	9.98	8.93	0	0	9.98	0	0	0
Linalool	50.22±2.38	13.22	11.84	0	13.22	0	0	0	0
(*E*,*E*)-2,4-Heptadienal	16.55±1.16	4.73	4.23	0	0	1.7	0.76	0	2.27
β-Cyclocitral	10.84±0.24	3.61	3.23	1.08	0.36	0	0	1.81	0.36
β-Ionone	5.94±0.40	57.11	51.14	17.13	5.71	0	0	28.56	5.71
Octanal	8.62±0.76	16.58	14.84	0	0	0	5.8	0.83	9.95
Decanal	3.62±0.04	1.2	1.07	0	0	0	0	0.24	0.96
Nonanal	10.53±0.47	3.01	2.69	0	0.6	0	0.3	0.3	1.81
Total	256.86	111.7*		18.44	19.89	11.68	7.09	32.19	22.41
%**				16.51	17.81	10.46	6.35	28.82	20.06

OAVi = Ci/Ti, where Ci is the content of compound i (μg/kg), and Ti is the threshold value of compound i (μg kg−1). * OVAt = ∑OAVi. ** % is proportion of the each odor OAV accounting for OAVt.

Table S10 The content and OAV-splitting of OAV>1 compounds in SB.

Compound	Ci ug/kg	OAVi	%	Split of odor characteristic (%)					
				Woody	Floral	Burnt	Green	Fruity	Fatty
Hexanal	65.83±1.01	2.2	1.82	0.22	0	0	0.22	0.44	1.32
Furaldehyde	77.38±3.35	9.67	8.01	0	0	9.67	0	0	0
Linalool	54.42±3.76	14.32	11.86	0	14.32	0	0	0	0
(*E*,*E*)-2,4-Heptadienal	16.22±1.09	4.63	3.83	0	0	1.67	0.74	0	2.22
β-Cyclocitral	11.32±0.15	3.78	3.13	1.13	0.38	0	0	1.89	0.38
β-Ionone	6.52±0.46	62.7	51.91	18.81	6.27	0	0	31.35	6.27
Octanal	9.89±0.28	19.02	15.75	0	0	0	6.66	0.95	11.41
Decanal	4.32±0.27	1.43	1.18	0	0	0	0	0.29	1.14
Nonanal	10.66±0.82	3.04	2.52	0	0.61	0	0.3	0.3	1.83
Total	256.56	120.79		20.16	21.58	11.34	7.92	35.21	24.57
%				16.69	17.87	9.39	6.56	29.15	20.34

Table S11 The content and OAV-splitting of OAV>1 compounds in SC.

Compound	Ci ug/kg	OAVi	%	Split of odor characteristic (%)					
				Woody	Floral	Burnt	Green	Fruity	Fatty
Hexanal	64.71±0.59	1.76	1.57	0.18	0	0	0.18	0.35	1.05
Furaldehyde	71.24±3.69	8.91	8.01	0	0	8.91	0	0	0
Linalool	24.93±0.10	6.56	5.9	0	6.56	0	0	0	0
(*E*,*E*)-2,4-Heptadienal	14.88±2.68	4.25	3.82	0	0	1.53	0.68	0	2.04
β-Cyclocitral	11.21±1.52	3.73	3.36	1.12	0.37	0	0	1.87	0.37
β-Ionone	6.64±0.34	63.83	57.43	19.15	6.38	0	0	31.92	6.38
Octanal	9.38±0.11	18.03	16.23	0	0	0	6.31	0.9	10.82

Decanal	3.95±0.33	1.31	1.18	0	0	0	0	0.26	1.05
Nonanal	9.7±2.12	2.77	2.49	0	0.55	0	0.28	0.28	1.66
Total	216.64	111.15		20.45	13.86	10.44	7.45	35.58	23.37
%				18.40	12.47	9.39	6.70	32.01	21.03

Table S12 The content and OAV-splitting of OAV>1 compounds in SD.

**Compound**	**Ci ug/kg**	**OAVi**	**%**	**Split of odor characteristic (%)**					
				**Woody**	**Floral**	**Burnt**	**Green**	**Fruity**	**Fatty**
Hexanal	44.21±1.41	1.47	2.02	0.15	0	0	0.15	0.29	0.88
2-Furaldehyde	48.52±4.62	6.07	8.34	0	0	6.07	0	0	0
(*E*,*E*)-2,4-Heptadienal	11.28±0.8	3.23	4.44	0	0	1.16	0.52	0	1.55
β-Cyclocitral	8.96±0.74	2.99	4.11	0.9	0.3	0	0	1.49	0.3
β-Ionone	7.14±0.75	54.23	74.49	16.27	5.42	0	0	27.12	5.42
Decanal	3.58±0.10	1.19	1.63	0	0	0	0	0.24	0.95
Nonanal	7.96±1.06	2.27	3.12	0	0.45	0	0.23	0.23	1.36
3-Methyl-butanal	10.84±0.7	1.35	1.85	0	0	0	0.2	0.61	0.54
Total	142.49	72.8		17.32	6.17	7.23	1.1	29.98	11
%				23.79	8.48	9.93	1.51	41.18	15.11
